# Teaching family medicine residents how to answer clinical questions using QUIPs

**Published:** 2013-09-30

**Authors:** Lisa Bishop, Norah Duggan, Heather Flynn

**Affiliations:** 1 School of Pharmacy and Faculty of Medicine, Division of Family Medicine, Memorial University of Newfoundland; 2 Faculty of Medicine, Division of Family Medicine, Memorial University of Newfoundland

## Abstract

**Background:**

“Questions in Practice” (QUIP) rounds are used to encourage residents to quickly find, evaluate, and incorporate information into clinical practice. It is an opportunity for residents to identify a clinical question, research the answer, present the evidence, and discuss how to apply it to practice. The value of using this method to teach residents has not been evaluated.

**Methods:**

A sampling of all first and second-year family medicine residents enrolled in the Memorial University Family Medicine program were invited to participate in the survey. The survey gathered information about the residents’ current experiences with answering clinical questions, their experience during QUIP rounds, and the value of an interdisciplinary approach.

**Results:**

The response rate was 91% (42/46). Medical websites (45%) and journal article indexes (34%) were most often used. Through QUIPs, 50% of the students identified new methods to retrieve answers, 80% considered it a useful learning experience, 75% had improved confidence, and clinical knowledge improved in 97%.

**Conclusions:**

Residents are familiar with many general sources of medical information, and QUIPs helped improve confidence in their knowledge and ability to answer questions. QUIPs appear to be a useful tool for teaching information resources and how to interpret and apply evidence to clinical situations.

## Introduction

It is estimated that clinicians generate 1 to 3 questions for every 3 patient visits, and 7 of every 10 questions go unanswered.^1^ This may be due to several things, including the time and effort needed to find the answer, the level of experience of the clinician, and the belief that there may not be an answer available.^1^–^2^ Obtaining accurate medical information is a key skill for answering questions pertaining to patient situations. There have been several studies examining the medical information needs of residents, the sources utilized by residents, and whether residents practice evidence-based medicine (EBM). In one study, residents in a university-based primary care internal medicine program were observed to determine the frequency and pursuit of medical information.^3^ It was determined that only 29% of identified questions were pursued, mostly by consulting textbooks, original articles or attending physicians. In another study, several barriers were identified as reasons why residents fail to answer clinical questions.^4^ Eight main themes emerged, including access to medical information, skills in searching information resources, clinical question tracking, time, clinical question priority, personal initiative, team dynamics, and institutional culture.

A recent meta-analysis captured the barriers that residents experience in applying EBM in daily practice.^5^ The primary barrier identified was limited available time, but other barriers included lack of motivation of residents, lack of knowledge and skill with respect to the EBM process, the potential negative influences of clinical supervisors and institutional barriers. Some solutions were suggested in some of the studies reported, but the outcome of these solutions was not examined. Although some of these studies have examined the behaviors of residents when they attempt to answer clinical questions, there is little information on effective methods used to improve family medicine residents’ ability to answer clinical questions in practice.

Two of the family medicine academic sites at Memorial University of Newfoundland (MUN) require the residents to participate in weekly QUIP rounds. QUIP rounds - otherwise known as “Questions in Practice” - is an opportunity for residents to identify a clinical question, research the answer, present the information they retrieve, and discuss how they would apply it to their practice. The residents present their QUIP findings to the academic family physicians, along with any other health professions who may attend the rounds (e.g., pharmacist, nurse).

The residents also attend EBM sessions throughout their residency, and QUIP rounds complement this by allowing residents to practically apply their skills to clinical situations. Although QUIP rounds have been ongoing for several years at these clinics, the value of using this method to teach family medicine residents how to answer clinical questions has not been evaluated. Since it is important that clinicians learn good information management skills on how to find, evaluate, and incorporate available evidence into their clinical practice, it would be useful to evaluate and communicate this method of teaching to other programs across the country.

The purpose of this study was to determine the opinions of family medicine residents about their experiences with answering clinical questions, the sources of information used to answer clinical questions, the value of QUIP rounds and the value of an interdisciplinary approach to QUIP rounds.

## Methods

### Setting and participants

The study was conducted in June 2008, with sampling of all first and second-year family medicine residents enrolled in the MUN family medicine program. A current list of residents was obtained from the family medicine program, and the survey was distributed to all the residents during an educational session. The survey along with a cover letter and self-addressed return envelope was distributed to the residents who were absent from the educational session.

### The instrument

The questionnaire was designed with input from all research team members. An existing survey that evaluated the benefit of QUIP rounds was not available in the literature. There were, however, a few studies that evaluated residents’ experiences with answering clinical questions and the resources used by residents to answer questions.^3^,^4^,^6^ The questions used in these surveys were taken into consideration when developing this questionnaire, along with articles on how to design questionnaires.^7^,^8^

The questionnaire gathered information about the residents’ current experiences with answering clinical questions including what sources they used to answer clinical questions, the residents’ experience during QUIP rounds including their searching, interpretation and application skills, and the value of interdisciplinary QUIP rounds. Demographic information about the person’s gender, year of birth, current year of residency, and where their medical degree was completed was also collected. The questionnaire was revised after pilot-testing on 2 academic family physicians at MUN. Opinions were captured through 5-point Likert scales, ranging from strongly agree to strongly disagree. For these questions, responses were collapsed to three categories of “agree” (included agree and strongly agree), “uncertain”, and “disagree” (included disagree and strongly disagree), reported as percentage of agreement or disagreement for each statement. Open responses were also captured.

### Ethics

Ethics approval was obtained through the Human Investigation Committee at Memorial University.

### Sample size and data analysis

A sample size calculation was not required given that all the family medicine residents in the program were surveyed. Data was entered into a SPSS (version 16.0) spreadsheet for analysis. Descriptive statistics were used for all questions.

## Results

All 1st and 2^nd^-year family medicine residents were surveyed. A total of 46 surveys were distributed, with 42 surveys returned, giving a response rate of 91%. The majority of those who completed the survey were female (*n* = 30, 71%) and the average age was 30 years (range 27–42). There was an almost equal split between first and second-year residents and the majority completed their medical degree at MUN (*n* = 27, 64%).

Sixty-seven percent (*n* = 28) of residents estimated generating more than 5 clinical questions per week. However, 69% (*n* = 29) of residents reported that they actually answered 5 or less questions per week. Eighty-eight percent (*n* = 37) reported that they felt comfortable answering clinical questions, 93% (*n* = 40) felt comfortable finding information to answer questions and 88% (*n* = 37) were able to find evidence-based answers to clinical questions.

They used a variety of different sources for finding answers but most commonly used medical information websites (*n* = 29) and journal article indexes (*n* = 18). The various sources they reported using are outlined in Table 1.

Of the 42 residents who completed the survey, 36 participated in QUIP rounds at some point during their residency. There was a variation in the estimated time that it took to prepare for QUIP rounds. Of the 36 residents who participated in QUIP rounds, 39% (*n* = 14) estimated that it took up to 30 minutes to prepare, 42% (*n* = 15) estimated that it took between 30 minutes to one hour, while the remainder thought that it took longer than an hour.

Table 2 summarizes 36 residents’ experiences with QUIP rounds including their searching skills, discussion with faculty members, other residents’ presentations, and their overall experience. Overall, 80% (*n* = 29) of residents who participated in QUIP rounds thought that it was a useful learning experience. QUIP rounds also had a perceived impact on patient care, as 73% (*n* = 26) thought that the information improved the care provided to patients, 75% (*n* = 27) thought that it improved their confidence, and 58% (*n* = 21) thought that it helped change their practice (Table 3).

Interdisciplinary QUIP rounds were experienced by ten residents. The pharmacist was present for nine residents and a nurse was present for one resident. The residents’ attitudes towards the participation of other health professionals were positive. The residents reported that different sources of information were introduced (*n* = 10), that the other health professionals contributed useful additional information (*n* = 9), that the additional information helped how they answered clinical questions (*n* = 6), and the QUIPs presented by other health professionals was relevant to their practice (*n* = 8).

The residents were also given the opportunity to provide open responses throughout the survey, with a total of 37 individual comments being made. Twenty-five comments were positive, with comments such as: a good learning experience; improved my knowledge; helped identify good sources of information; will use this method of finding information in practice; should be continued as part of the rotation. Two residents reported that QUIPs were not useful, and 6 residents made comments around how the utility of QUIPs was lessened because of multiple learning activities. Four stated that they never participated in QUIP rounds.

## Discussion

Introducing QUIP rounds into the resident’s teaching enables residents to use their searching and retrieving skills in daily practice. There are many barriers identified in the literature to explain why it is difficult for clinicians to answer clinical questions, including insufficient time, knowledge, and lack of searching and critical appraisal skills.^5^,^9^

Medical information is changing virtually on a daily basis, with the introduction of new medications, new studies and new guidelines, making it crucial for physicians to keep up to date with the ever-changing information.^1^ Ninety-seven percent of the residents who responded to our survey thought that their knowledge was improved through the use of QUIP rounds, and almost three quarters felt that it improved their confidence and the care they provided to their patients. Half of them also reported that it helped identify new methods of retrieving answers to questions, which is a key skill in helping maintain their information retrieval abilities. By making family medicine residents more comfortable with searching for answers to questions, they will be more likely to retain this skill and knowledge base when they are practicing physicians.

Other health professionals can also contribute to residents’ learning by introducing a different perspective and discussing different resources for answering clinical questions.^10^ The survey suggests that other health professionals expose residents to a different approach to answering questions while at the same time increasing their knowledge. Since only a few residents were exposed to other health professionals in this setting, it would be useful to further explore this interdisciplinary approach in contributing to the learning experience of the residents in a primary health care setting.

There were several limitations to this study. Even though there was a very good response rate of 91%, there are a fixed number of residents in the program. At the time the survey was distributed, 46 residents were enrolled in the program, consisting of an equal split of residents who just completed either their 1^st^ or 2^nd^ year of the program. Since the residents complete different rotations at different times, not all of the residents’ experiences were the same, and not all were exposed to other health professionals during these rounds. As well, the residents completed QUIP rounds at different times throughout their residency, thus increasing the possibility of recall bias.

Another limitation is that the survey was not validated. Since QUIP rounds is a novel approach to teaching, an existing survey available that asked questions about this method of teaching did not exist in the literature. The survey was developed taking into consideration questions that were used in other surveys, and was tested on academic faculty members before being distributed to the residents. Since there were a fixed number of residents who could complete the survey, testing the survey on a portion of them would have decreased the number able to complete the survey.

The comments from the residents helped solidify the benefit of QUIP rounds. They indicated that it was a useful experience that helped introduce additional sources of information. Workload was a concern, as they participate in multiple activities such as teaching diaries, case conferences, academic half day and weekend rounds, all of which reduce clinical time with patients. As such, it is important that the number of activities is balanced so that it complements the learning process. Based on responses from residents, it was suggested that at the beginning of the rotation the faculty review the types of evidence available for finding good evidence-based answers in a timely manner.^11^ The residents should be encouraged to answer questions efficiently, by selecting practical questions to present and using resources that are reliable and provide good evidence.

## Conclusions

Participation in QUIP rounds is a useful learning experience for residents. With medical information constantly changing, it is very challenging for clinicians to keep up to date. Residents need the tools to find and apply current information in their practice and they often lack confidence in their clinical knowledge. Involvement in QUIP rounds helped improve their confidence in their knowledge and their ability to provide patient care. Teaching family medicine residents the tools to use resources, interpret data, and apply the information to clinical situations will ultimately transpire into practicing family physicians with the skills to provide a greater level of care to their patients.

## Figures and Tables

**Table 1:** Information sources used (*n* = 42)

Source*	Number (%)
Medical information websites	55 (45)
Journal article indexes	41 (34)
General internet search engine	10 (8)
Colleague/Preceptors	8 (7)
Medicine texts	7 (6)
Other	1 (1)

* may select more than 1 source within each category

**Table 2:** Evaluation of QUIP rounds experience (*n* = 36)*

Statement	Missing *n* (%)	Disagree *n* (%)	Uncertain *n* (%)	Agree *n* (%)
It was easy to identify a clinical question to answer.	0	3 (8)	6 (17)	27 (75)
My searching skills improved throughout the rotation.	0	6 (17)	14 (39)	16 (44)
I identified new methods of retrieving answers.	0	7 (19)	11 (31)	18 (50)
I identified new sources of information to answer questions.	1 (3)	11 (31)	9 (25)	15 (42)
The discussion with faculty members during QUIP rounds was useful.	1 (3)	3 (8)	2 (6)	29 (81)
The information presented by other residents and students was useful.	0	2 (6)	2 (6)	32 (89)
Overall it was a useful learning experience.	0	3 (9)	4 (11)	29 (80)
I would recommend QUIP rounds to other residents.	0	6 (17)	5 (12)	25 (69)

* percentages may not add up to 100 due to rounding

collapsed categories are reported (disagree = disagree/strongly disagree; agree = agree/strongly agree)

**Table 3:** Impact of QUIP rounds on patient care decisions (*n* = 36)*

Statement	Disagree *n* (%)	Uncertain *n* (%)	Agree *n* (%)
The information improved the care I provided patients.	2 (6)	8 (22)	26 (73)
The information improved my confidence in the care I provided to patients.	2 (6)	7 (19)	27 (75)
The information improved my knowledge regarding the topic.	1 (3)	0	35 (97)
The information was helpful and not confusing.	1 (3)	6 (17)	29 (81)
My communication with patients improved about the topics discussed.	5 (14)	13 (36)	18 (50)
The information discussed helped change my practice.	3 (8)	12 (33)	21 (58)

* Percentages might not add to 100 owing to rounding

collapsed categories are reported (disagree = disagree/strongly disagree; agree = agree/strongly agree)

## References

[1] McConaghy JR (2006). Evolving medical knowledge: Moving toward efficiently answering questions and keeping current. Prim Care Clin Office Pract.

[2] Ely JW, Osheroff JA, Chamblis ML, Ebell MH, Rosenbaum ME (2005). Answering physicians’ clinical questions: Obstacles and potential solutions. J Am Med Inform Assoc.

[3] Green ML, Ciampi MA, Ellis PJ (2000). Residents’ medical information needs in clinic: Are they being met?. Am J Med.

[4] Green ML, Ruff TR (2005). Why do residents fail to answer their clinical questions? A qualitative study of barriers to practicing evidence-based medicine. Acad Med.

[5] van Dijk N, Hooft L, Wieringa-de Waard M (2010). What are the barriers to residents’ practicing evidence-based medicine? A systematic review. Acad Med.

[6] Leahy N, Sheps J, Tracy CS, Nie JX, Moineddin R, Upshur REG (2008). Family physicians’ attitudes toward education in research skills during residency. Can Fam Physician.

[7] Choi BCK, Pak AWP (2005). A catalog of biases in questionnaires. Prev Chronic Dis.

[8] VanGeest JB, Johnson TP, Welch VL (2007). Methodologies for improving response rates in surveys of physicians: A systematic review. Eval Health Prof.

[9] Ebell MH (2009). How to find answers to clinical questions. Am Fam Physician.

[10] Iwanowicz SL, Marciniak MW, Zeolla MM (2006). Obtaining and providing health information in the community pharmacy setting. Am J Pharm Educ.

[11] Kendall S (2008). Evidence-based resources simplified. Can Fam Physician.

